# Sidechain Metallopolymers with Precisely Controlled Structures: Synthesis and Application in Catalysis

**DOI:** 10.3390/polym14061128

**Published:** 2022-03-11

**Authors:** Rui Qu, Hongyi Suo, Yanan Gu, Yunxuan Weng, Yusheng Qin

**Affiliations:** 1 College of Chemistry and Chemical Engineering, Yantai University, Yantai 264005, China; quruiyantai@163.com (R.Q.); suohongyi92@163.com (H.S.); joanne1028@ytu.edu.cn (Y.G.); 2 Beijing Key Laboratory of Quality Evaluation Technology for Hygiene and Safety of Plastics, College of Chemistry and Materials Engineering, Beijing Technology and Business University, Beijing 100048, China

**Keywords:** multi-metallic catalyst, sidechain, metallopolymer, living and controlled polymerization, precise control of polymer structure

## Abstract

Inspired by the cooperative multi-metallic activation in metalloenzyme catalysis, artificial enzymes as multi-metallic catalysts have been developed for improved kinetics and higher selectivity. Previous models about multi-metallic catalysts, such as cross-linked polymer-supported catalysts, failed to precisely control the number and location of their active sites, leading to low activity and selectivity. In recent years, metallopolymers with metals in the sidechain, also named as sidechain metallopolymers (SMPs), have attracted much attention because of their combination of the catalytic, magnetic, and electronic properties of metals with desirable mechanical and processing properties of polymeric backbones. Living and controlled polymerization techniques provide access to SMPs with precisely controlled structures, for example, controlled degree of polymerization (DP) and molecular weight dispersity (*Đ*), which may have excellent performance as multi-metallic catalysts in a variety of catalytic reactions. This review will cover the recent advances about SMPs, especially on their synthesis and application in catalysis. These tailor-made SMPs with metallic catalytic centers can precisely control the number and location of their active sites, exhibiting high catalytic efficiency.

## 1. Introduction

Metalloenzymes with metal ions as cofactors account for approximately one-third of the known enzymes [1]. The properties of catalytic metals in proteins are modulated by the primary and secondary coordination sphere, which are related to ligands and active site environment, respectively [2]. Owning to the delicate and diverse structures, metalloenzymes can catalyze numerous reactions with physiological importance, such as O_2_ reduction and N_2_ fixation, with high reactivity and selectivity [3].

Many researchers have focused on using natural enzymes for industrial applications in consideration of their amazing properties in nature. However, the application of native enzymes out of physiological environments faces a great challenge because of their low operational stability, extreme sensitivity of catalytic activity to environmental conditions, incompatibility with organic solvents, difficulties in recovery and recycling, and high costs in preparation and purification [4,5,6]. Thus, artificial enzymes have been developed for mimicking the catalytic functions of natural enzymes and, at the same time, overcoming the limitations of natural ones [7,8]. Although many attempts have been made to reproduce the structures and functions of enzymes, many more studies are still needed to develop synthetic materials equivalent to natural enzymes in terms of structure, catalytic efficiency, specificity, selectivity, etc. [9].

One of the most popular topics for artificial enzymes is the construction of multi-metallic catalysts, inspired by cooperative multi-metallic activation in enzyme catalysis [10,11,12,13,14]. In many enzymes, two or more metal centers in the active site are able to activate both nucleophilic and electrophilic reactants, leading to improved kinetics and higher selectivity. Researchers have proven that dual/multiple activation catalysts can accomplish higher efficiencies than conventional monofunctional catalysts in terms of reactivity, substrate selectivity and, potentially, cost-efficiency [15,16,17,18,19]. For bimetallic or trimetallic catalysts, well-designed ligands with low molecular weight could fully fulfill the cooperative interaction between metals [20,21,22]. However, for multi-metallic catalysts with more metal centers, synthetic polymers as macromolecular ligands are more appropriate because of their high loading capacity of metals.

Early research about metallic polymeric catalysts focused on metal ions or metal complexes supported on cross-linked polymers, such as cross-linked polystyrene [23,24], which are mostly heterogenous. The polymeric catalysts showed excellent recoverability, higher solvent compatibility, thermal stability, and lower oxygen and moisture sensitivity compared to homogeneous catalysts [25]. However, it is difficult for these supported catalysts to precisely control the number and location of their active sites, which made it difficult to construct multi-metallic catalysts with higher activity and selectivity [26].

Metallopolymers with metals in the sidechains, also named as sidechain metallopolymers (SMPs), have become quite popular during the past few years [27]. The appeal of these hybrid compounds is attributed to their ability to combine the catalytic, magnetic, and electronic properties of metals with desirable mechanical and processing properties of polymeric frameworks [27]. The development of living and controlled polymerization techniques provides access to linear polymers with precisely controlled structures, which make it possible for ordered spatial arrangement of metal centers. In many studies, linear polymers were crosslinked or folded into metal-containing nanoparticles, which offers many opportunities for the development of artificial enzyme-mimic catalysts, showing both high catalytic activity and specificity [28,29]. Actually, a polymer chain can be more than a component of a larger assembly. By carefully engineering at the molecular level, a single polymer chain can behave as an individual functional object with its own characteristics and function [30].

**Scheme 1:**
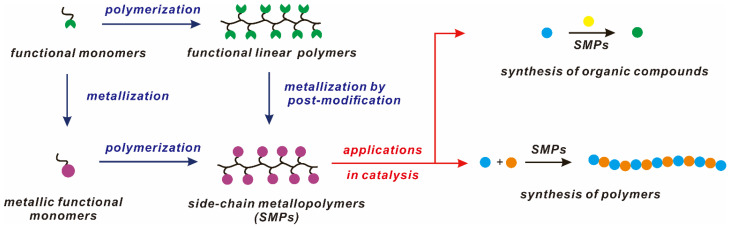
General strategies for the preparations of SMPs and their applications in catalysis.

This review will cover the recent advances in SMPs as multi-metallic catalysts. We intend to illustrate several flexible and efficient synthetic methods to construct tailor-made metallopolymers. The applications of these metallopolymers as catalysts are reviewed in relation to synthesis for organic compounds and polymers (Scheme 1).

## 2. Synthetic Methods of Linear Polymers with Precisely Controlled Structures

Living polymerization is an ideal chain growth process without chain termination and irreversible chain transfer, which represents an excellent strategy to precisely control the molecular weight and the molecular weight distribution of linear polymers [31,32,33].

Atom transfer radical polymerization (ATRP) and reversible addition-fragmentation chain transfer (RAFT) polymerization, as controlled radical polymerizations, are widely used to prepare well-defined linear polymers with precisely controlled structures. ATRP is a versatile technique based on the use of a catalytic system consisting of a metal center complexed by a ligand. This catalytic system is able to abstract a halogen from an alkyl halide initiator, creating a radical that initiates the polymerization [34]. In terms of the types of polymerizable monomers, RAFT is currently the most versatile technique for performing controlled radical polymerization. In contrast to ATRP, this system relies on the rapid exchange between active and dormant chains. The chain end of a dormant chain bears a thiocarbonyl-thio moiety, which is a chain transfer active species [35]. Nearly all monomers applicable to ATRP could be polymerized well by RAFT with narrow molecular weight distribution. Actually, depending on the activating substituents, the whole gamut of vinyl monomers, including functional ligands [36,37,38,39] and functional (metal) complexes [18,40,41], can be polymerized in a controlled manner by RAFT, leading to polymers with low dispersity.

Ring-opening metathesis polymerization (ROMP) is also a particularly powerful method for the synthesis of well-defined linear polymers with precise molecular weight control and complex polymeric architectures [32,42]. ROMP is a chain-growth polymerization process by which cyclic olefins are converted to polymers with an unsaturated main chain catalyzed by transition metal-alkylidene complex, such as ruthenium (Ru) complex (Grubbs catalysts) and molybdenum (Mo) complex (Schrock catalysts) [43]. Norbornene is one of the most common cyclic olefins used in ROMP, which can be readily functionalized by large and chemically active organic or inorganic moieties. Many functional polynorbornenes bearing metal complexes have been generated and further applied in catalysis and biodetection [44,45,46,47]. Polynorbornenes with functional groups in sidechains could be further connected with other functional molecules, such as metals or metal complexes, by post-modification [17,48]. In addition, other cyclic olefins, including 1-cyclobutene [49], cyclohexene [50], cyclooctene [51] and 1,5-cyclooctadiene [52,53], have also been successfully used to build functional polymers. The polymerization of norbornene derivatives can also be performed via addition polymerization (AP), also called vinyl/addition polymerization, which leads to a saturated structure of main chains [54]. Although AP is more thermodynamically favorable than ROMP, it is more sensitive to substituents in the monomers. Highly active late transition-metal nickel (Ni)- or palladium (Pd)-catalysts are required for the polymerization of norbornenes with bulky substituents [55].

Living and controlled polymerizations allow for the preparation of well-defined SMPs with a predetermined degree of polymerization (DP) and low dispersity (*Đ*), and further provide the polymers with controlled architectures and functionalities, which makes it possible for SMPs to be used as highly efficient catalysts for a series of chemical reactions.

## 3. Preparation of SMPs

Metals or metal complexes can be prepared as polymerizable monomers, and further polymerized into SMPs via living and controlled polymerizations, which seems to be the best method for constructing SMPs for the high loading capacity and efficiency of metals. However, for catalytic molecules with complicated structures or large molecular sizes, polymerizations may meet difficulties because of the steric hindrance effect. Post-modification of side groups in linear polymers is a complementary method to the first approach, which was suitable for metal-complexes meeting difficulties in the preparation into polymerizable monomers or metal-complexes with large molecular sizes. In addition, metals or metal complexes can also act as crosslinkers to induce the folding of linear polymers to form nanoparticles.

### 3.1. Polymerization of Metal-Containing or Ligand-Containing Monomers

**Figure 1:**
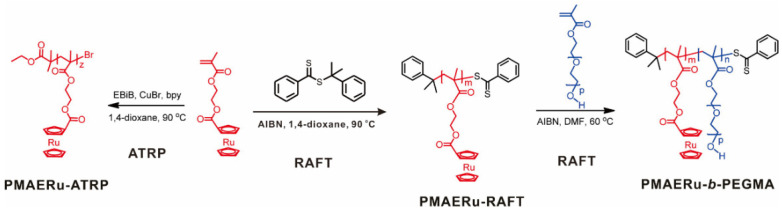
Synthesis of ruthenocene-containing homopolymers and diblock copolymers by RAFT and ATRP. Reprinted from Ref. [40] with permission. Copyright 2013 American Chemical Society.

Well-designed metal-containing vinyl monomers can be polymerized into SMPs by controlled radical polymerization [56,57]. Tang and coworkers successfully prepared ruthenocene (Ru)-containing methacrylate polymers by ATRP and RAFT polymerization [40]. By using the Ru-containing homopolymer as a macro-RAFT agent and a macroinitiator, diblock copolymers were synthesized via successful chain extension, as shown in Figure 1. The *Đ*s of the metallopolymers remained at low values of 1.15–1.21. The calculated DP of the polymers could reach over 50 and showed a good consistency with the theoretical value. The synthesis and characterization of ruthenocene-containing methacrylate monomer and polymers in this work paved the way to develop a variety of metal-containing polymers with different functionalities and architectures, which have the promise for new applications in the field of organometallic polymers.

**Figure 2:**
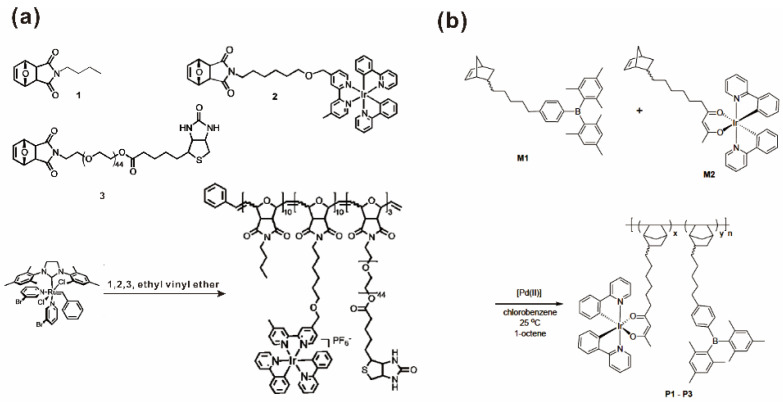
(**a**) Synthesis of the iridium-containing block copolymers via ROMP. Reprinted from Ref. [47] with permission. Copyright 2012 American Chemical Society; (**b**) Synthesis of the iridium-containing block copolymers via AP. Reprinted from Ref. [59] with permission. Copyright 2012 American Chemical Society.

Similarly, metal-containing norbornene-based monomers can be polymerized into SMPs by ROMP or AP. Sleiman and coworkers used ROMP to generate a series of iridium-containing block copolymers with the goal of using their self-assembled micelles as luminescent markers in biodetection assays [46,47,58]. Norbornene-based ROMP monomers 1, 2 and 3 with different functions were designed and prepared (Figure 2a). Monomer 1 as the hydrophobic block was used to induce the self-assembly and to comprise the micelle core. Monomer 2 contained iridium (Ir)(III) complex as luminescent moiety, which was the core functional part for the further application in biodetection assays. Monomer 3 contained poly(ethylene glycol) (PEG) as a water-soluble block to form the corona of the micelle and as well as a biotin molecule as a biorecognition element that was able to bind to target groups. Block copolymer was synthesized by sequential polymerization of monomers 1–3, respectively, using ROMP with the third generation ruthenium-based Grubbs catalyst [47]. In another work, vinyl-type polynorbornene copolymers containing sidechain iridium moieties were prepared by copolymerization of norbornene monomers bearing triarylborane and an iridium emitter catalyzed by a cationic Pd(II) catalyst (Figure 2b) [59].

**Figure 3:**
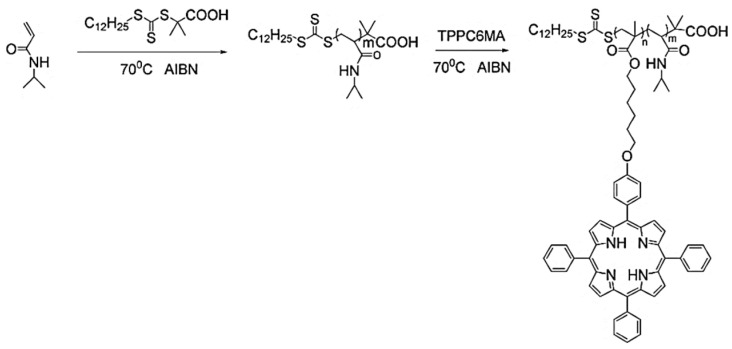
Synthesis of PNIPAM-*b*-PTPPC6MA block copolymer. Reprinted from Ref. [62] with permission. Copyright 2010 Royal Society of Chemistry.

Porphyrins are widely used as photosensitizers in photodynamic therapy (PDT) and efficient catalysts in enzymatic reactions as well as in polymer synthesis when coordinated with metals [60,61]. Well-designed porphyrin-based vinyl monomers can be polymerized into homopolymers or block copolymers by RAFT polymerization. Zhang and coworkers prepared amphiphilic PNIPAM-*b*-PTPPC6MA block copolymers from porphyrin-containing monomers using RAFT polymerization, as shown in Figure 3 [62]. The DP of the porphyrin monomer ranged from 5 to 37 in the block copolymers, which had a narrow *Đ* of 1.12–1.23, indicating a good control of the polymerization. The block copolymers could further self-assemble into a variety of morphologies ranging from micelles to vesicles, which exhibited a high singlet oxygen quantum yield and had potential application for PDT.

Sidechain polymers prepared from ligand-containing monomers, such as porphyrin-based monomers [18] or salen-based monomers [63], could be further coordinated with metals and used as catalysts with excellent performance.

### 3.2. Post-Modification of Side Groups

**Figure 4:**
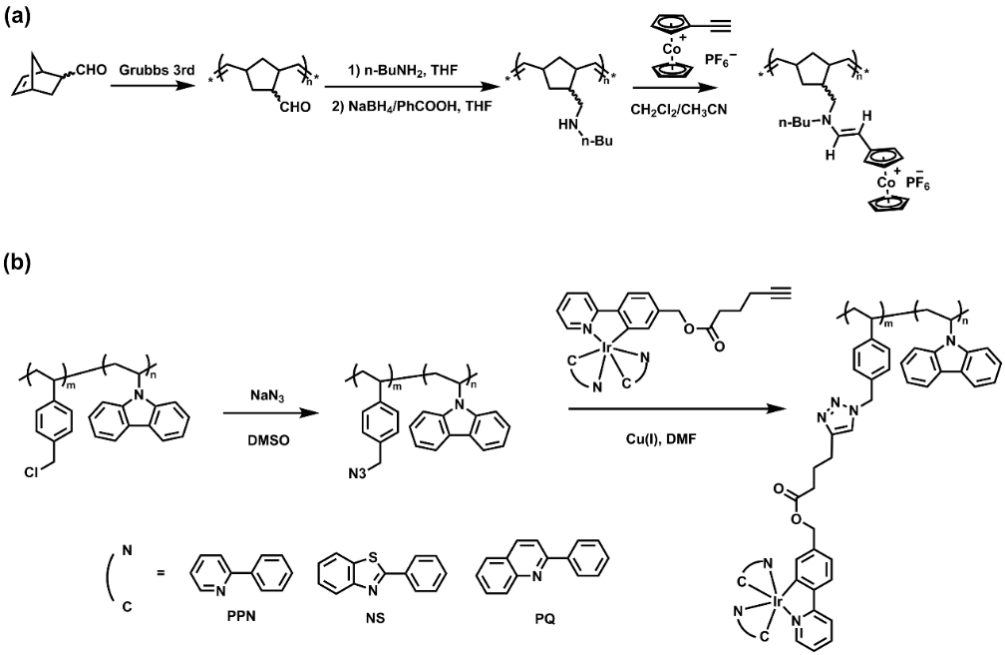
(**a**) Post-modification of norbornene-based polymers by amino-yne reaction yielding cobalticenium-enamine-functionalized polymers. Reprinted from Ref. [64] with permission. Copyright 2014 American Chemical Society; (**b**) functionalization of the copolymers via alkyne-azide “click” chemistry. Reprinted from Ref. [65] with permission. Copyright 1996 Royal Society of Chemistry.

Post-modification of side groups in linear polymers is also widely used in the preparation of SMPs. Metal complexes were combined with the side groups of the polymers by reactions with high efficiency, for example, “click” reactions, including alkyne-azide reaction and amino-yne reaction, Michael addition reaction, and carboxyl-amine reaction. In Figure 4a, a norbornene derivative functionalized with a secondary amine group was polymerized by ROMP using the 3rd generation of Grubbs catalyst, followed by hydroamination of ethynyl cobalticenium, yielding the cobalticenium-enamine-functionalized norbornene polymer with the yield of 97% [64]. In another work, as shown in Figure 4b, *n*-vinyl carbazole or styrene was copolymerized with para-(chloromethyl)styrene by radical polymerization, and then the chloromethylated copolymers were converted to the corresponding azides in 90–95% yields. Alkyne-functionalized iridium further reacted with the copolymers via “click” chemistry to yield the desired functionalized copolymers, with complete conversion of the azide groups to the 1,4-disubstituted 1,2,3-triazoles supported by NMR spectra [65].

**Figure 5:**
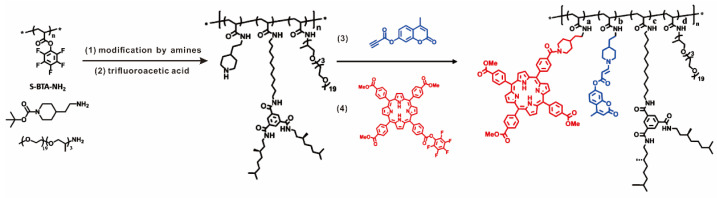
Synthetic routes using post-modification method for porphyrin-containing poly(pentafluorophenyl acrylate) with multiple functional groups. Reprinted from Ref. [66] with permission. Copyright 2015 American Chemical Society.

Compared with metallopolymers prepared from metal-containing or ligand-containing monomers, post-modification of linear polymers appears to be more operable to prepare polymers carrying more than one kind of ligand or functional molecule. Meijer and coworkers developed a general post-modification strategy of poly (pentafluorophenyl acrylate) (pPFPA) as a versatile method to rapidly access functional polymers [66], as shown in Figure 5. The piperidine-containing polymers were further connected with a different ratio of carboxyl acid functionalized porphyrin derivative and alkyne-functionalized coumarin derivative, forming functional polymers with photosensitizing properties.

### 3.3. Metals as Cross-Linkers

**Figure 6:**
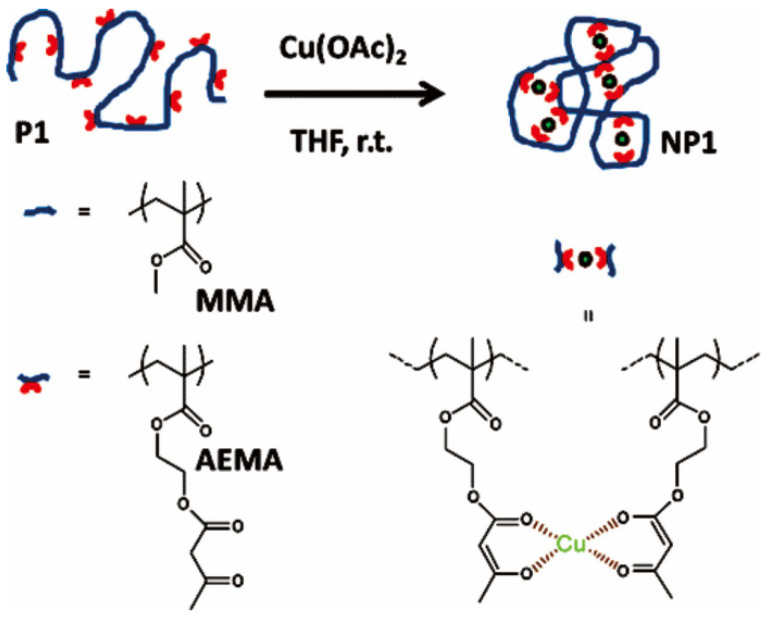
Synthetic route of the Cu(II)-crosslinking polymers assisted by coordination interaction of Cu(II)-*β*-ketoester. Reprinted from Ref. [71] with permission. Copyright 2014 American Chemical Society.

Metals or metal complexes can act as cross-linkers to induce the folding of linear polymers under highly diluted conditions via intra-chain interactions, either non-covalent or covalent intra-chain interactions, which leads to nanoparticles, also called single-chain nanoparticles [28,67,68]. Recently, Cu(II)-mediated intramolecular cross-linking of polymers has attracted much attentions [36,69,70,71,72]. Pomposo and coworkers prepared metallo-folded nanoparticles based on copolymers containing methyl methacrylate (MMA) and 2-(acetoacetoxy) ethyl methacrylate (AEMA), which were prepared by RAFT polymerization and were further folded by the intrachain interaction between *β*-ketoester of AEMA and Cu(II), as shown in Figure 6 [71]. The nanoparticles were formed under mild conditions, in tetrahydrofuran (THF) at room temperature by using Cu(OAc)_2_ as reagent at high dilution conditions (polymer concentration 1 mg/mL). This work also paved the way for the easy and efficient construction of other metallo-folded polymers (based on Pd, Ni, Co, Fe, Mn, or Mo) approaching the substrate specificity of natural enzymes for a variety of organic reactions. Other copper-crosslinking polymers with different types of polymers such as aspartate-containing polyolefins [72] and poly(imidazole)s [36] were also prepared, which could be used as catalysts for “click” chemistry.

## 4. Applications of SMPs in Catalysis

SMPs with precisely controlled structures offer many possibilities for the development of multi-metallic catalytic systems from the following aspects: (i) precise control of the number and location of catalytic sites; (ii) improved stability and processability of metals on the polymer chains; (iii) high catalyst concentration in confined space; (iv) cooperative effect of multi-site catalytic groups. Two types of reactions are reviewed here: synthesis of organic compounds and polymers, as shown in Table 1.

### 4.1. Synthesis of Organic Compounds

#### 4.1.1. Oxidation of Sulfide

**Figure 7:**
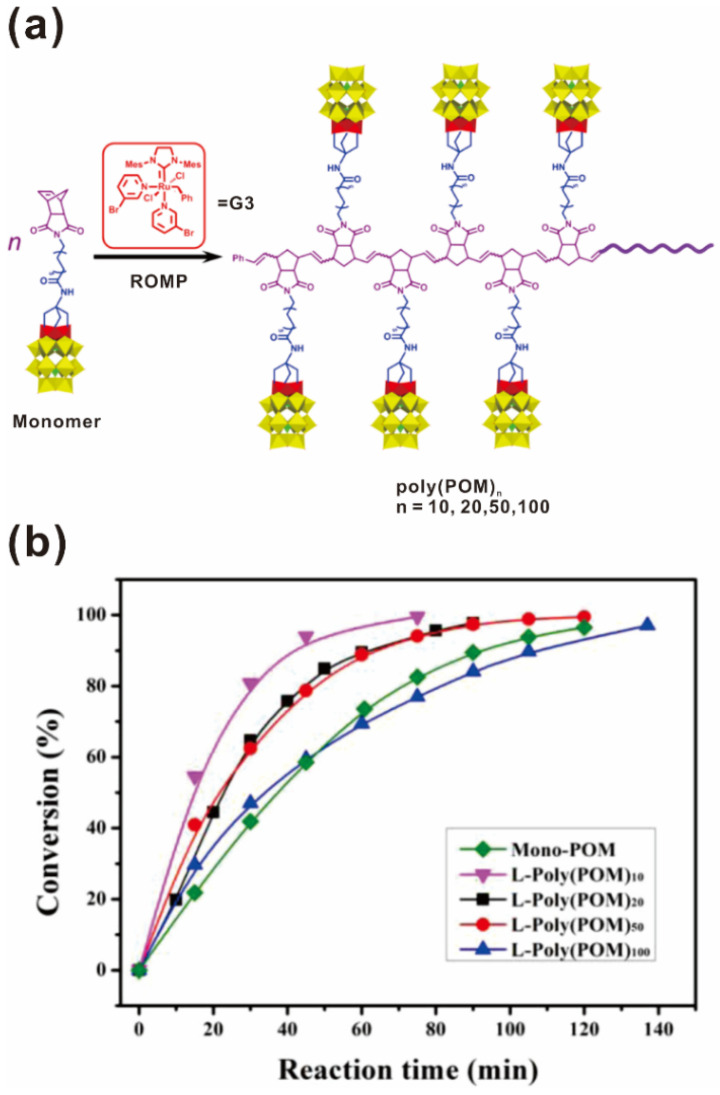
(**a**) Synthetic route of the poly(POM)s; (**b**) Conversions of tetrahydrothiophene oxide (THTO) in the oxidation of tetrahydrothiophene (THT) catalyzed by Mono-POM and L-Poly(POM)s. Reprinted from Ref. [45] with permission. Copyright 2014 American Chemical Society.

Polyoxometalates (POMs), as a class of anionic clusters of early-transition metals in their highest oxidation states, have been extensively investigated as photocatalysts or electrocatalysts, mainly involved in acid and oxidation catalysis [83,84]. Wang and coworkers integrated POMs into polymer matrices to create novel POM-containing polymers possessing the unique properties of POM clusters and the favorable processability of polymers [45,73,74,75]. Poly(POM)s were synthesized from POM-containing norbornene monomers by ROMP in the presence of a Grubbs catalyst under mild conditions with yields of nearly 100% in a living and controllable manner, as shown in Figure 7a [45]. The *Đ*s of the poly(POM)s were between 1.07 and 1.15, indicating well-controlled polymerization characteristics (Table 2). This work further indicated that the special chemical activity and steric hindrance of the POM cluster do not impede polymerization of the POM-containing monomer or lead to undesirable side reactions. Unlike pure POM clusters, poly(POM)s can be processed from solutions to form thin films, which was contributed to the organic polynorbornene backbone. The poly(POM)s were further used to catalyze the oxidation of the sulfide, tetrahydrothiophene (THT), which exhibited better catalytic reactivity than POM monomers, as shown in Figure 7b. Over 95% THT was converted to THTO in 90 min and no sulfone was observed. The catalytic reactivity decreased when the DP was increased above 10, probably due to the polymer chain coils in the dilute solution.

#### 4.1.2. Hydroformylation of Alkene

**Figure 8:**
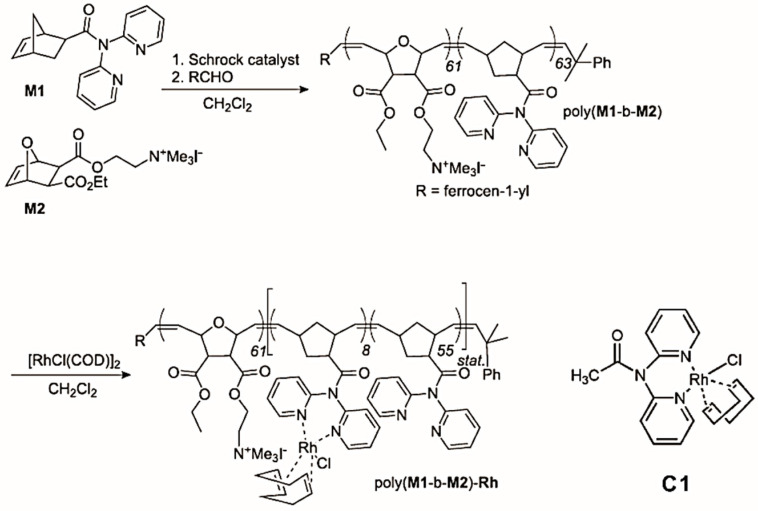
Synthesis of poly(M1-*b*-M2) and of the micellar catalyst poly(M1-*b*-M2)-Rh and the homogeneous analogue CH_3_CON(Py)_2_RhCl(COD) (C1). Reprinted from Ref. [76] with permission.

The hydroformylation reaction, starting from alkenes, dihydrogen and carbon monoxide to yield *n*-aldehydes, as well as the corresponding regioisomers, is one of the most important industrial processes, with a global production of several million tons [85,86]. Rh-based catalysts are the preferred catalytic systems for their high performance at low temperatures [87]. Buchmeiser, Blechert and coworkers reported an amphiphilic block copolymer modified by a Rh-*N,N*-dipyrid-2-ylacetamide-based catalyst on the sidechain and its application in hydroformylation reactions [76]. The amphiphilic block copolymer poly(M1-*b*-M2) was prepared from two norbornene-based monomers M1 and M2 using a Schrock catalyst. M1 contains a chelating ligand and M2 contains a quaternary ammonium moiety. The polymer was further loaded with Rh(I) via reaction with (RhCl(COD))_2_ (COD = cycloocta-1,5-diene) to yield poly(M1-*b*-M2)-Rh, with 12% of the dipyrid-2-ylamide ligands involved in complex formation (Figure 8). Poly(M1-*b*-M2)-Rh and the homogeneous analogue C1 [86] were further used as catalysts for the hydroformylation of 1-octene. The *n:iso* ratio of the catalytic reaction catalyzed by poly(M1-*b*-M2)-Rh was higher than the one obtained with C1 in toluene, i.e., 1.5 vs. 0.9 (Table 3), which showed that the micellar catalyst favored the formation of the *n*-aldehyde. This enhanced selectivity was attributed to the large percentage (88%) of free dipyrid-2-ylamide ligands, as well as to the high concentration of reactants within the micelle, apparently suppressing *β*-elimination in the alkyl-metal species. Compared with the homogeneous catalysts, the micellar catalysts were more easily separated from the products, leading to lower metal contamination of the products, as well as to the possibility of reuse.

#### 4.1.3. Copper-Catalyzed Azide-Alkyne Cycloaddition

**Figure 9:**
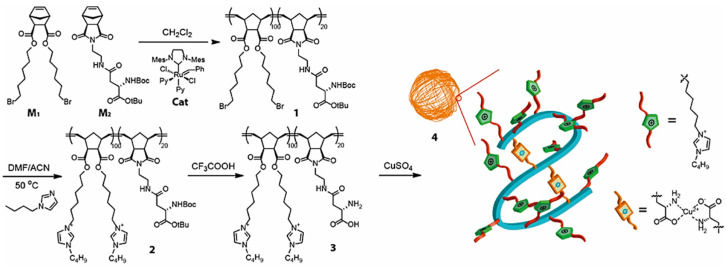
Synthetic route of copper-containing metal-organic nanoparticles (Cu-MONPs). Reprinted from Ref. [69] with permission. Copyright 2016 American Chemical Society.

Copper-catalyzed azide-alkyne cycloaddition (CuAAC) is the most common “click” reaction that allows researchers to link together two organic, bioorganic, or other functional molecular fragments [88,89]. The large quantity of metal catalyst used in CuAAC “click” reaction and its difficulty of complete removal remain the main problems to be solved [90]. Zimmerman and coworkers prepared copper-containing metal-organic nanoparticles (MONPs) via Cu(II)-mediated intramolecular cross-linking of aspartate-containing polyolefins, as shown in Figure 9, which served as highly efficient catalysts for alkyne-azide “click” reactions, yielding the desired 1,4-adducts at low parts per million catalyst levels [69]. The catalytic activity of Cu-ONPs was tested in the Cu(I)-catalyzed reaction between phenylacetylene and benzyl azide, as shown in Table 4. 1.0–10 ppm of Cu-ONPs were employed with respect to benzyl azide and using sodium ascorbate to reduce Cu(II) to Cu(I) in the MONPs in situ. In the presence of 2.5–10 ppm Cu-MONPs, with respect to benzyl azide at 50 °C, reactions achieved completion in 24 h, with the 1,4-triazole as the only product. In the absence of Cu(I) or in the presence of CuSO_4_, only a low conversion of the starting material to product was observed, with a mixture of 1,4- and 1,5-triazoles, indicative of an uncatalyzed reaction. Indeed, the nanoparticles have low toxicity and low metal loadings, making them convenient, green catalysts for alkyne-azide “click” reactions in water.

#### 4.1.4. Reduction of Nitrophenol

**Figure 10:**
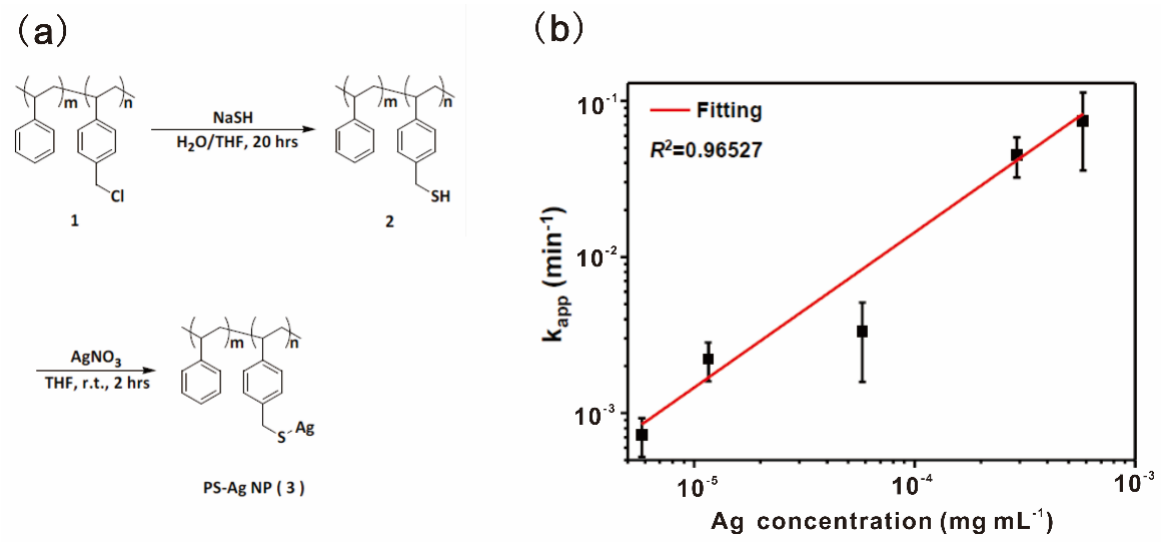
(**a**) The synthetic route of PS-AgNPs; (**b**) The apparent rate constant *k*_app_ as a function of Ag in L-PS-AgNPs. Reprinted from Ref. [77] with permission. Copyright 2012 Royal Society of Chemistry.

4-aminophenol (4-AP) is a potent intermediate for manufacturing many analgesic and antipyretic drugs. 4-AP could be prepared by hydrogenation of 4-nitrophenol (4-NP), which is also an efficient method for the removal of nitrophenols from wastewater and contaminated aquifers [91]. Metal nanoparticles as catalysts for reduction of 4-NP to 4-AP with high-performance have long been developed [92,93,94]. The limited stability and aggregation for colloidal nanoparticles are typically the major issues in catalysis. Liu, Huang and coworkers reported a highly efficient polystyrene-based nano-silver-containing polymer (PS-AgNPs) as a catalyst for the reduction of 4-NP [77]. The soluble PS-AgNPs were synthesized based on post-modification of AgNPs in the polymer sidechains, as shown in Figure 10a. Entanglements of polystyrene chains restricted the aggregation of AgNPs in the polymer sidechains and made it possible to form stable nano-silver domain sizes. The linear relationship between rate constant *k*_app_ and Ag concentrations in PS-AgNPs with low Ag content (L-PS-AgNPs) supported that AgNPs in L-PS-AgNPs were evenly distributed and exhibited excellent dispersion stability (Figure 10b). The catalytic performance of PS-AgNPs with high Ag content (H-PS-AgNPs) was comparable to the microreactor, including the core of AgNPs/SiO_2_ and polymer shell, as shown in Table 5. PS-AgNPs provided a new strategy to confine the size domain of metal nanoparticles immobilized on polymers, which was relatively simple compared to microreactors with core/shell designs.

#### 4.1.5. Hydrolytic Kinetic Resolution of Epichlorohydrin

**Figure 11:**
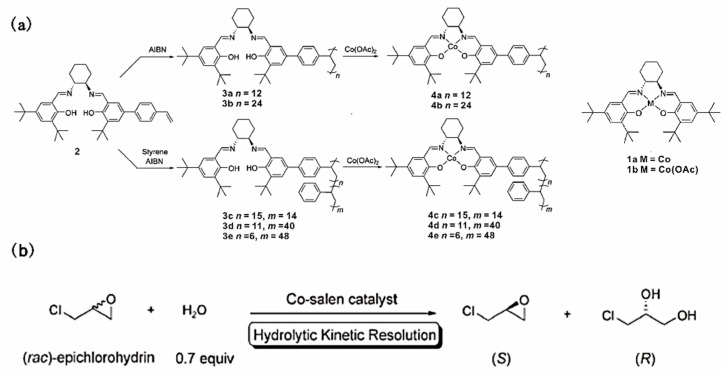
(**a**) Synthesis of poly(styrene)-supported Co-salen complexes and molecular Co-salen complex; (**b**) The reaction of hydrolytic kinetic resolution of epichlorohydrin. Reprinted from Ref. [63] with permission. Copyright 2005 The Royal Society of Chemistry.

Chiral salen complexes are a family of catalysts for a series of important asymmetric organic transformations, including the epoxidation of olefins, the hydrolytic kinetic resolution (HKR) of epoxides, hetero Diels–Alder reactions and conjugate addition reactions [96,97,98,99]. Weck, Jones and coworkers prepared a series of polymer-supported salen catalysts formed by free radical polymerizations of an unsymmetrical monostyryl-substituted salen monomer, as shown in Figure 11 [63]. The salen moieties were attached to the polymer chain in a flexible, pendant fashion and, hence, possessed a high degree of flexibility and accessibility. The copolymer-supported Co-salen complexes showed a better catalytic performance (>99% enatiomeric excess of the remaining epichlorohydrin, 54% conversion) compared to the homopolymeric analogues and the small molecule Co-salen complex, as shown in Table 6. The better catalytic performance of the copolymer was attributed to the greater Co-salen complex mobility compared with homopolymer, which made the catalytic sites more accessible to the reactants. In addition, the copolymers with more flexible polymer backbones would increase the possibility of intramolecular cooperation between cobalt catalytic sites. The soluble poly(styrene)-supported catalysts could be easily recovered by precipitation after the catalytic reactions; the reaction rates were slightly reduced after three round cycles.

### 4.2. Synthesis of Polymers

#### 4.2.1. Olefin Polymerization

In the field of catalytic olefin polymerization, it is assumed that proximate metal centers would significantly influence the monomer enchainment and the chain transfer kinetics, which, in turn, leads to unique catalytic consequences, such as higher activity, enhanced branching, higher olefin incorporation, and increased molecular weight as compared to their mononuclear analogues [78,100,101]. Thus, in the past two decades, great efforts have been devoted to design binuclear or multinuclear complexes for cooperative olefin polymerization [100,102,103]. It is shown that conjugated or adjacent metal active centers can mutually influence the electron density of the metal centers as well as protection of the active centers, thus, enhancing monomer trapping and illustrating the positive consequences.

**Figure 12:**
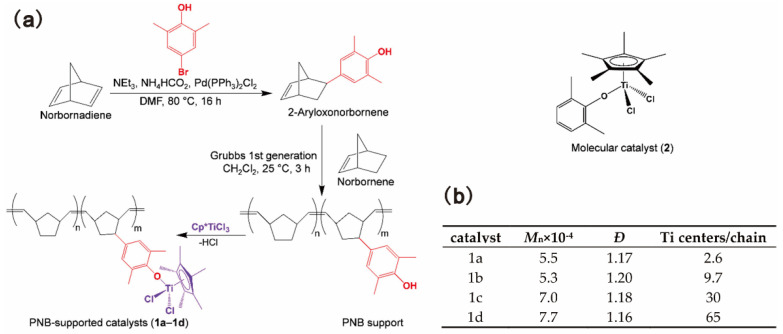
(**a**) Synthetic route of PNB-supported half-titanocene catalysts and the structure of the molecular catalyst; (**b**) Summary of PNB-Supported catalyst synthesis. Reprinted from Ref. [17] with permission. Copyright 2019 American Chemical Society.

Taniike and coworkers prepared a series of polynorbornene (PNB)-supported half-titanocene catalysts bearing different numbers of Ti centers per PNB chain [17,78]. Monodispersed PNB chains bearing a predefined number of ancillary donor ligands at the sidechain was synthesized by ROMP and, further, grafted half-titanocene complexes to afford structurally well-defined supported catalysts, as shown in Figure 12a. This synthetic route enabled precise control of the number of Ti centers per PNB chain, as shown in Figure 12b, which made it possible for the systematic investigation of the synergistic effect among multiple active centers. On activation with a sterically less frustrated borate system, the PNB-supported catalysts exhibited higher activity than that of the corresponding molecular analogues. The activity increased with the increase in the number of Ti centers per chain of the catalysts. The highest activity was observed for 1d bearing 65 Ti centers, which was nearly twice the activity of the molecular catalyst 2, as shown in Table 7 [17]. The catalysts 1a and 1b bearing a smaller number of Ti centers produced PE with higher molecular weight, probably due to the suppression of chain transfer reactions under low activity conditions.

**Figure 13:**
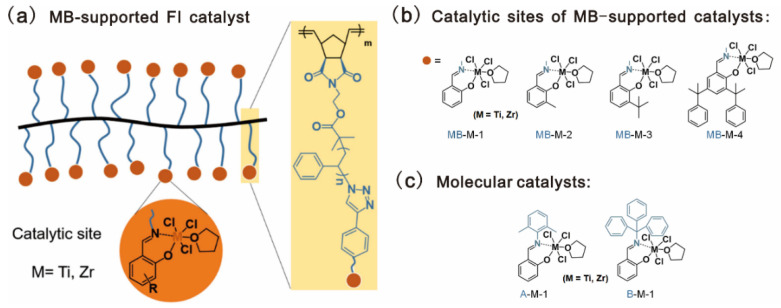
Chemical architectures of: (**a**) MB-supported FI catalysts; (**b**) catalytic sites MB-M-X, where M and X refer to metals (Ti or Zr) and salicylaldehyde derivatives, respectively; (**c**) molecular catalysts. Reprinted from Ref. [79] with permission. Copyright 2021 American Chemical Society.

A molecular bottlebrush (MB), composed of a linear backbone grafted with polymeric sidechains, was also used as the catalyst support for ethylene polymerization. Compared with conventional linear polymers, MBs display many unique features, such as the nanoscale size, higher persistent length of the single chain, and reduced overlap of neighboring chains [104]. Chen and coworkers reported a poly(norbornene-*graft*-styrene) (PNB-*g*-PS)-supported mono(phenoxy-imine) metal complex for ethylene polymerization, as shown in Figure 13a [79]. PNB-*g*-PS-Br was prepared via grafting-through ROMP of the norbornenyl macromonomer, which was readily prepared by ATRP. Post-modification reactions of PNB-*g*-PS-Br were performed to incorporate phenoxy-imine type ligands in PS sidechains, which were then coordinated with early transition metals to generate a series of MB-supported mono(phenoxy-imine) catalysts. Four types of phenoxy-imine ligands and two metals (Ti and Zr) were used for the catalysts, as shown in Figure 13b. Improved catalytic activities were observed for the catalysts bearing bulky substituents orthogonal to the phenolic oxygen in the ligands. The highest values are 369 and 461 kg PE mol^−1^ h^−1^ for the Ti and Zr catalysts, with a cumyl group, as shown in Table 8. The obtained polyethylenes (PEs) have high crystallinities and tunable molecular weights ranging from 80 to 2000 kDa.

#### 4.2.2. CO_2_/Epoxides Copolymerization

Most epoxide ring-opening reactions follow a cooperative pathway, in which one metal serves as a Lewis acid for epoxide activation and another as a counterion for nucleophilic attack. Enhanced activities and enantioselectivities have been observed for bimetallic complexes compared with their monometallic counterparts [105,106,107]. It is supposed that building a multi-metallic catalyst composed of molecularly well-defined active centers will increase the local catalyst concentration and amplify the metal-metal cooperativity to a larger extent than bimetallic catalysts.

**Figure 14:**
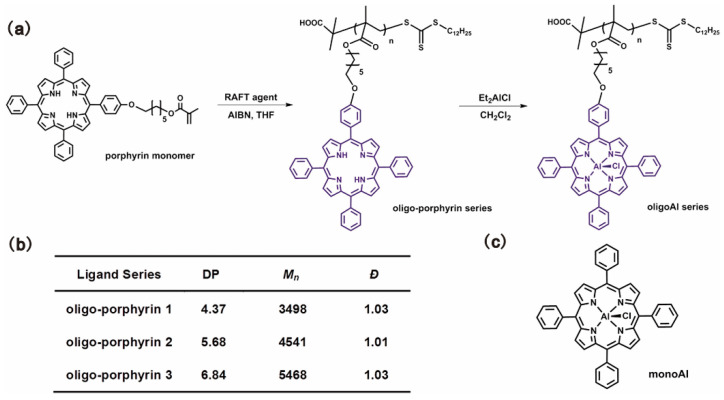
(**a**) Synthesis procedure from porphyrin monomers to the final oligoAl catalysts. (**b**) Synthetic results of the oligoporphyrin series. (**c**) Chemical structure of the monoAl catalyst. Reprinted from Ref. [18] with permission. Copyright 2019 American Chemical Society.

**Figure 15:**
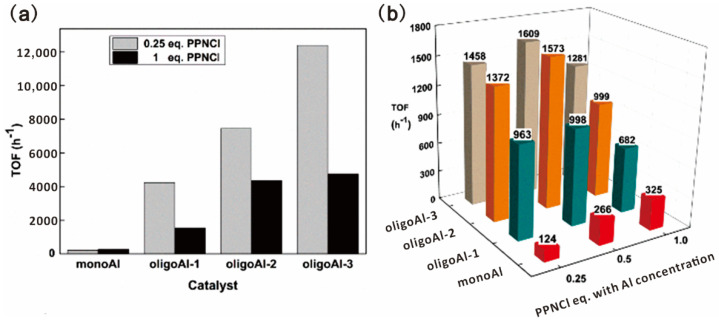
Catalytic performance of the oligoAl series and monoAl with different amounts of PPNCl. TOFs for (**a**) PO homopolymerization and (**b**) PO/CO_2_ copolymerization. Reprinted from Ref. [18] with permission. Copyright 2019 American Chemical Society.

Wang and coworkers reported a series of oligomer catalysts flexibly bearing aluminum (Al)(III) porphyrin complexes in the sidechains as multi-metallic catalysts for epoxide ring-opening reactions to obtain CO_2_-based polymers [18,80,81]. Oligomerization of porphyrin methacrylate was carried out by RAFT polymerization with precisely controlled DP and narrow *Đ*, as shown in Figure 14 [18]. Homopolymerization of PO catalyzed by the oligoAl series and monoAl were firstly investigated. In combination with PPNCl as a cocatalyst, the oligoAl catalysts showed much higher rates than monoAl, and the turnover frequency (TOF) value continuously increased as more Al centers were anchored in one catalyst chain, as shown in Figure 15a. For copolymerization of PO and CO_2_, similar tendencies in the relationship among the activity, the number of anchored Al centers, and the ratio of added cocatalyst, were observed, as shown in Figure 15b. In addition, the oligoAl series catalysts exhibited preeminent enhancement at extremely low loadings, high polymer selectivity at high temperature, and the capability to produce high-molecular-weight CO_2_-based polycarbonate, owing to the multisite cooperativity in the “confined transfer” chain growth scenario and 100% utilization of Al sites in homogeneous catalytic behavior.

For bimetallic catalysts used in CO_2_/epoxides copolymerization, researchers have proven that the metal distance was closely related to polymer selectivity (polycarbonate/polyether) [108] and catalytic activity [109]. The influence of metal distance on catalytic performance is also worth exploration for multi-metallic catalysts. The metal distance in SMPs can be regulated by designing main-chains with different lengths. However, for living and controlled polymerizations (ATRP, RAFT, ROMP, etc.), the main-chains are usually singles forms, which are difficult to regulate. Thus, new polymerization methods that can produce SMPs with diverse main-chain structures are needed for deep exploration about the catalytic mechanism of multi-metallic catalysts.

#### 4.2.3. ATRP

**Figure 16:**
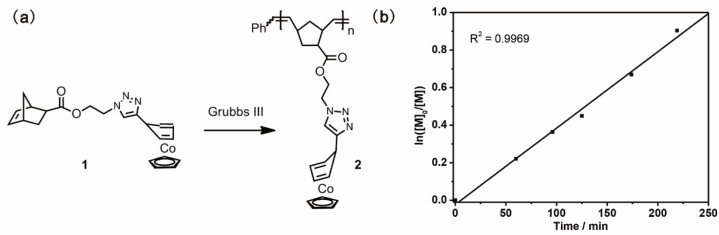
(**a**) Synthesis of Co(I)-containing polymer; (**b**) Semilogarithmic plots of polymerization of MMA using polymer **2** as a heterogeneous catalyst. Reprinted from Ref. [82] with permission. Copyright 2014 The Royal Society of Chemistry.

Previously, Wang and coworkers reported an exo-substituted *η*^4^-cyclopentadiene CpCo(I) complex that could be used as a homogenous molecular catalyst in Co(I)-mediated ATRP [110]. The molecular catalyst was soluble in either monomer or solvent, which resulted in polymers contaminated with a color from the catalyst. Tang and coworkers reported a sidechain polymer containing cyclopentadienyl-cobalt(I)-1,3-cyclopentadiene as a heterogeneous macromolecular catalyst for ATRP, which could obtain polymers without color from the catalyst [82]. The macromolecular catalyst was prepared from an 18-e cobalt(I)-containing norbornene (monomer **1**) by ROMP using the 3rd generation of Grubbs catalyst, as shown in Figure 16a. The obtained polymer **2** was soluble in organic solvents such as dichloromethane (DCM), chloroform, and tetrahydrofuran (THF), but was insoluble in monomers (i.e., MMA, styrene) and the solvent (i.e., toluene) involved in ATRP. The living characteristic of the polymerization was confirmed by the linear semilogarithmic plot, and the polymerization rate was comparable to those of molecular catalysts reported in the literature (Figure 16b). The catalyst could be easily removed from the products by filtration because of its heterogeneity in the reaction media. Different from the light green color of the products obtained using monomer **1** as the catalyst, the obtained PMMA and polystyrene do not have any color from the catalyst. 

## 5. Conclusions and Outlooks

In this review, we discuss sidechain metallopolymers (SMPs) from preparation methods to applications in catalysis. Inspired by the structures of natural metalloenzymes, polymers are used to function as scaffolds for metals, just like the role of polypeptides in enzymes. SMPs with ordered structures and diverse functional groups can be prepared by various living and controlled polymerization techniques, and metal complexes can be connected to the polymers either before or after polymerizations. The obtained SMPs can be successfully applied as effective multi-metallic catalysts for both organic compound synthesis and polymer synthesis. Therefore, SMPs have proven to be excellent models for multi-metallic catalysts for their precise control of the number and location of catalytic sites. There are still some important issues about SMPs deserving further explorations, for example, the influence of the sidechain properties, especially the chain length and the chemical structure, on the catalytic performance of SMPs. Polymers with the same main chain and various sidechains should be prepared and studied as the base for SMPs.

Although great efforts have been made to construct metallopolymers with precise structures as multi-metallic catalysts, there is still a long way to go for endowing a linear polymer chain with special functions as natural enzymes. In peptides, the repeat unit sequence is well-defined, although as many as 20 amino acid comonomers are involved in the polymerization [111]. For copolymers prepared by radical polymerization, the positions and sequences of these functionalities are totally random and not uniform among polymer chains [30]. Thus, the sequence of polymers is recognized as the next structural factor that can be controlled precisely in polymerizations to express advanced functions of polymers as observed in nature [111,112,113,114,115]. Many efforts have been made to construct sequence-ordered polymers, for example, sequence-regulated radical polymerization with a metal-templated monomer to achieve polymers with AB [116] or ABA [111] alternating sequences. These sequence-ordered polymers bring more possibilities for multi-metallic catalysts carrying more than one kind of metals, which could be available for constructing metallic catalysts with alternating metals to achieve unexpected catalytic performance. More challenging works about sequence-ordered polymers prepared by sequence-regulated polymerizations are urgently needed and are expected to enrich the multi-metallic catalyst system and artificial enzymes.

## Figures and Tables

**Table 1:** Reaction types catalyzed by different SMPs.

No.	Reaction	Reaction Type	Catalytic System	Preparation Method	Refs.
1	oxidation of sulfide	synthesis of organic compounds	poly(POM)s	PMM ^1^	[45,73,74,75]
2	hydroformylation of alkene		Rh-SMPs	post-modification	[76]
3	CuAAC		Cu-Nanoparticles	metals as crosslinkers	[36,69,72]
4	reduction of nitrophenol		Ag-SMPs	post-modification	[77]
5	HKR of epichlorohydrin		Co-SMPs	PLM ^2^	[63]
6	olefin polymerization	synthesis of polymers	Ti/Zr-SMPs	post-modification	[17,78,79]
7	CO_2_/epoxide copolymerization		Al-SMPs	PLM	[18,80,81]
8	ATRP		Co-SMPs	PMM	[82]

^1^ PMM: Polymerization from metal-containing monomer. ^2^ PLM: Polymerization from ligand-containing monomer.

**Table 2:** Molar ratios, molecular weights, and *Đ*s of the monomer and hybrid polymers. Reprinted from Ref. [45] with permission. Copyright 2014 American Chemical Society.

Catalysts	[M]/[G3]	*M*_n_, Theo (kDa)	*M*_w_, SEC (kDa)	*Đ*
Mono-POM		5.8	5.5	1.16
L-Poly(POM)_10_	10/1	58.3	59.4	1.09
L-Poly(POM)_20_	20/1	116.7	116.7	1.07
L-Poly(POM)_50_	50/1	291.6	270.9	1.15
L-Poly(POM)_100_	100/1	583.3	774.9	1.15

**Table 3:** Results for the hydroformylation of 1-octene ^1^. Reprinted from Ref. [76] with permission.

No.	Catalyst	Solvent	TON ^2^	TOF	*n:iso*
1	poly(M1-*b*-M2)-Rh	water	3800	1200	1.5
2	C1	toluene	4500	2700	0.9

^1^ catalyst:substrate ratio = 1:5000, t = 4 h, T = 70 °C. ^2^ based on the aldehydes formed.

**Table 4:** Results for “Click” reactions of benzyl azide and phenylacetylene with Cu-MONPs or CuSO_4_. Reprinted from Ref. [69] with permission. Copyright 2016 American Chemical Society.

Cu-MONP Level (ppm)	Cu Level (ppm)	NaAsc	t (h)	T (°C)	Yield (%)
1.0	10	+	24	50	58
2.5	25	+	24	50	>99
5.0	50	+	24	50	>99
10	100	+	24	50	>99
2.5	25	+	72	25	>99
0	25 ^1^	+	336	25	9 ^2^
2.5	25	−	336	25	9 ^2^
0	0	−	24	50	9 ^2^
0	25	+	24	50	9 ^2^

^1^ The corresponding copper level was reached by adding CuSO_4_ solution. ^2^ Yield of the 1,4-isomer. The crude product also contained approximately the same amount of the 1,5-isomer for these entries.

**Table 5:** Different AgNP-based catalysts with equivalent Ag content and their corresponding apparent rate constants.

Catalyst	Solvent	Ag Concentration (mg mL^−1^)	*k*_app_ (min^−1^)	Ref.
AgNPs/SiO_2_	THF/water 9/1 (*v*/*v*)	5.4 × 10^−4^	0.0382	[95]
MC(AgNPs/SiO_2_) ^1^		5.1 × 10^−4^	0.2089	
L-PS-AgNPs		5.8 × 10^−4^	0.0797	[77]
H-PS-AgNPs		5.0 × 10^−4^	0.3880	

^1^ microcapsule denoted as MC.

**Table 6:** Results of the hydrolytic kinetic resolution of epichlorohydrin. Reprinted from Ref. [63] with permission.

No.	Catalyst	t (h)	Conv. (%)	ee ^1^ (%)
1	1a	1.0	49	93
2	4a	1.0	47	81
3	4b	1.0	48	83
4	4c	1.0	50	90
5	4d	1.0	54	99
6	4e	1.0	54	99

^1^ ee refered to the enatiomeric excess of the remaining epichlorohydrin.

**Table 7:** Ethylene polymerization results using PNB-supported half-titanocene catalysts and the molecular catalyst under the same polymerization condition. Reprinted from Ref. [17] with permission.

No.	Catalyst	Activity ((kg of PE)/((mol of Ti) h))	*M*_w_ × 10^−5^	*Đ*
1	2	16,400 ± 390	5.7	2.5
2	1a	22,100 ± 50	6.5	2.9
3	1b	22,800 ± 100	6.2	2.9
4	1c	28,600 ± 110	4.9	2.3
5	1d	29,800 ± 200	5.8	2.6

**Table 8:** Ethylene polymerization using the molecular and the MB-supported catalysts. Reprinted from Ref. [79] with permission. Copyright 2021 American Chemical Society.

No.	Catalysts	*w*_m_ (%) ^1^	Activity ^2^	*M*_w_ (kDa)	*Đ*
1	A-Ti-1		9	2295	1.69
2	B-Ti-1		11	1704	2.88
3	MB-Ti-1	0.97	41	2051	3.35
4	MB-Ti-2	0.99	100	298	3.41
5	MB-Ti-3	0.85	50	568	1.76
6	MB-Ti-4	0.78	369	83	2.20
7	A-Zr-1		15	1309	2.88
8	B-Zr-1		23	4095	1.52
9	MB-Zr-1	2.50	16	260	4.24
10	MB-Zr-2	3.00	22	188	13.20
11	MB-Zr-3	2.53	460	372	6.72
12	MB-Zr-4	2.63	461	496	4.84

^1^ Metal weight percentage (*w*_m_) in the supported catalysts analyzed by ICP-MS. ^2^ Activity in unit of kg PE mol^−1^ h^−1^.

## Data Availability

Not applicable.
